# Supplementary figures and images for: The Diagnostic Value of ACSL1, ACSL4, and ACSL5 and the Clinical Potential of an ACSL Inhibitor in Non-Small-Cell Lung Cancer
Source: Cancers (Basel). 2024 Mar 16;16(6):1170. doi: 10.3390/cancers16061170 (PMC10969076; doi:10.3390/cancers16061170)

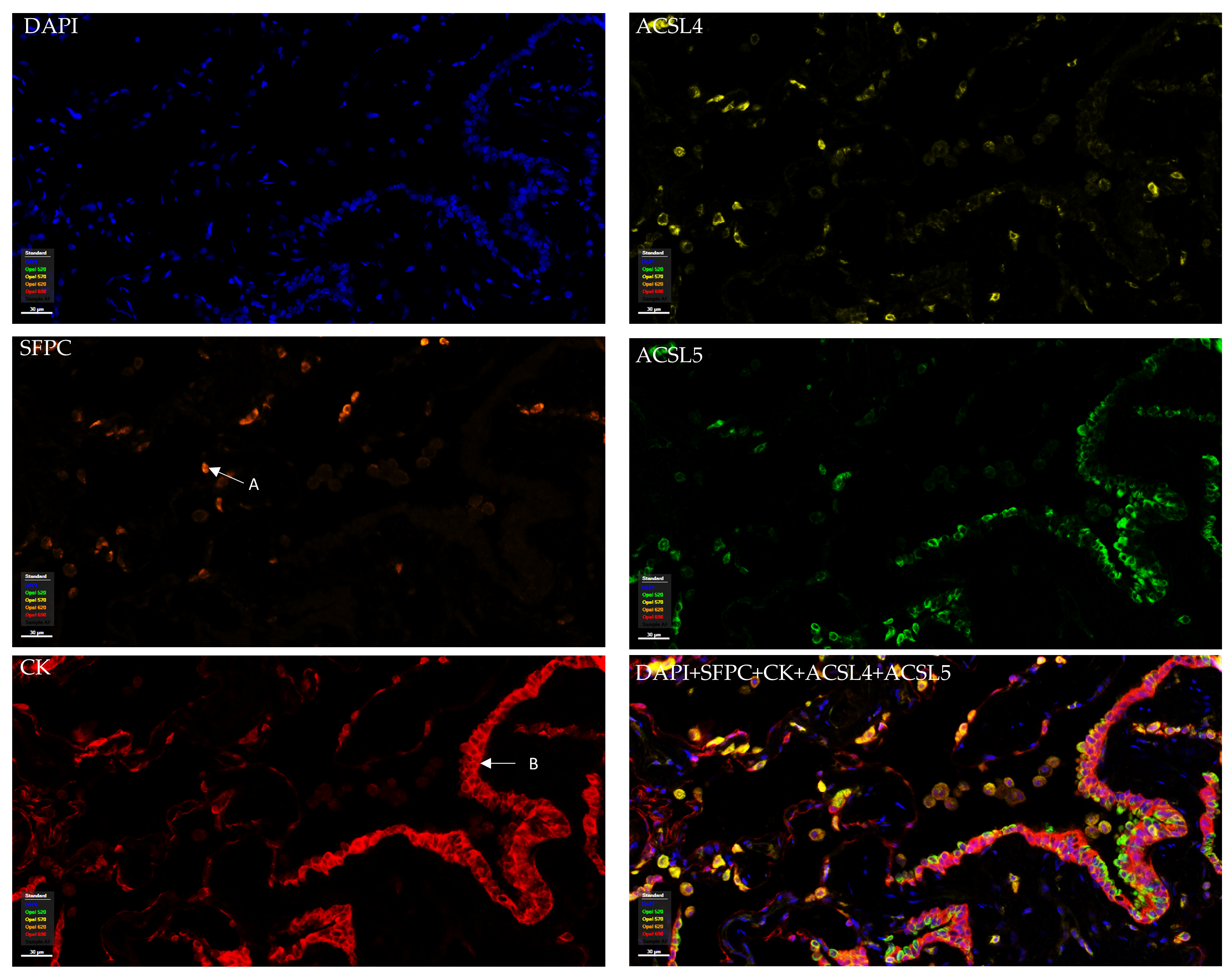

Supplement: Supplementary file 1 [file cancers-16-01170-s001.zip › Figure S1.TIF]

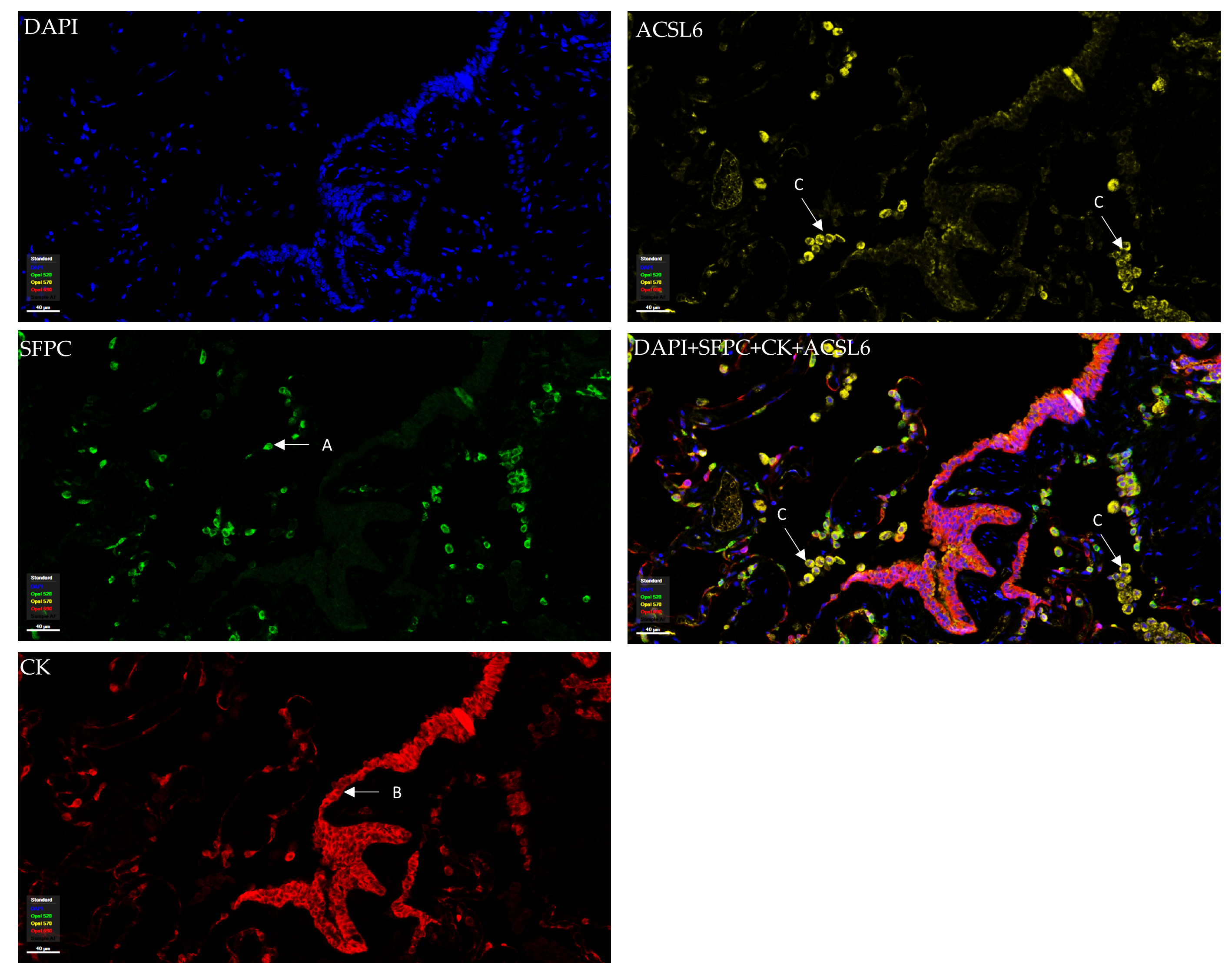

Supplement: Supplementary file 1 [file cancers-16-01170-s001.zip › Figure S2.TIF]

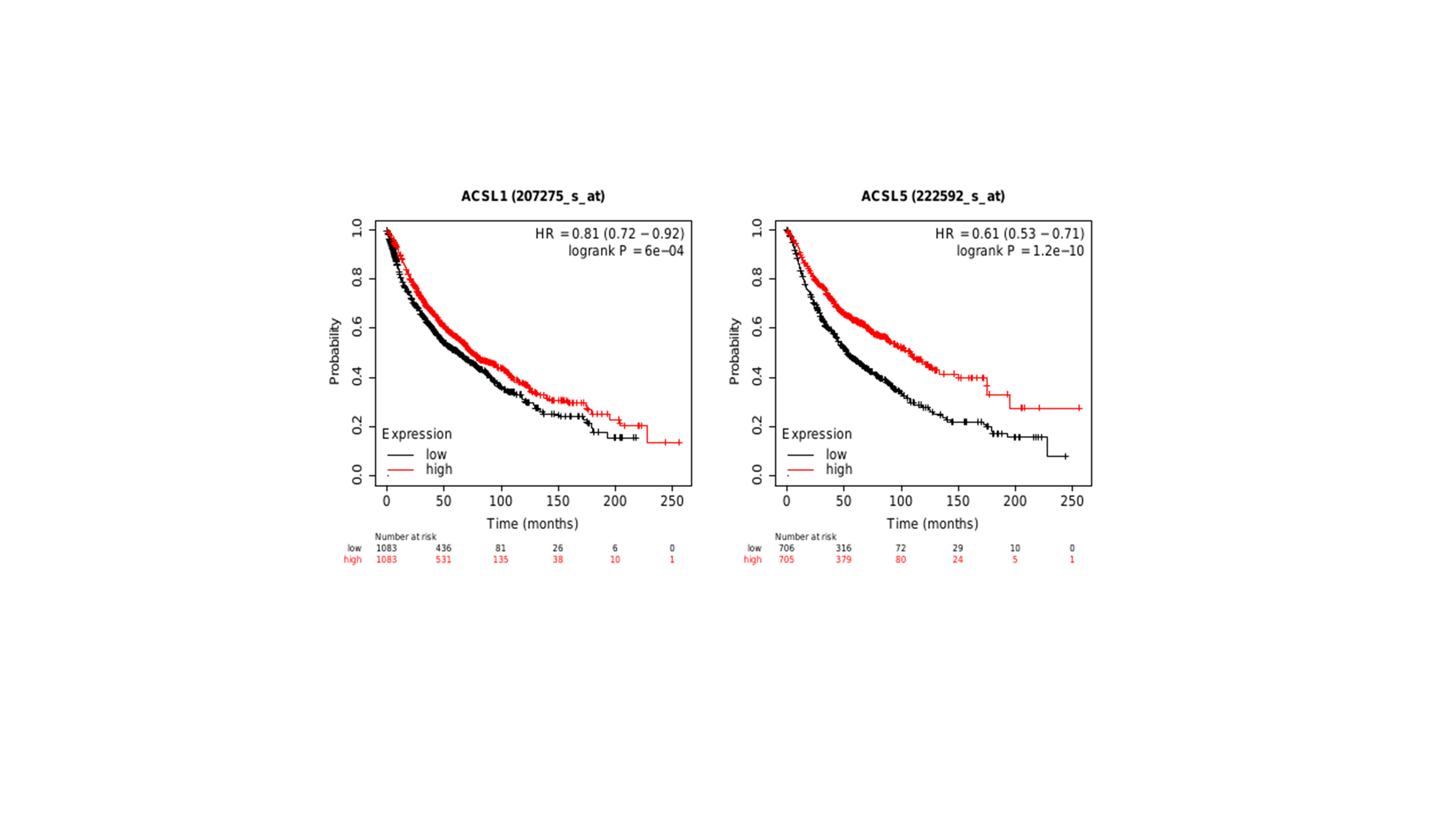

Supplement: Supplementary file 1 [file cancers-16-01170-s001.zip › Figure S3.tif]

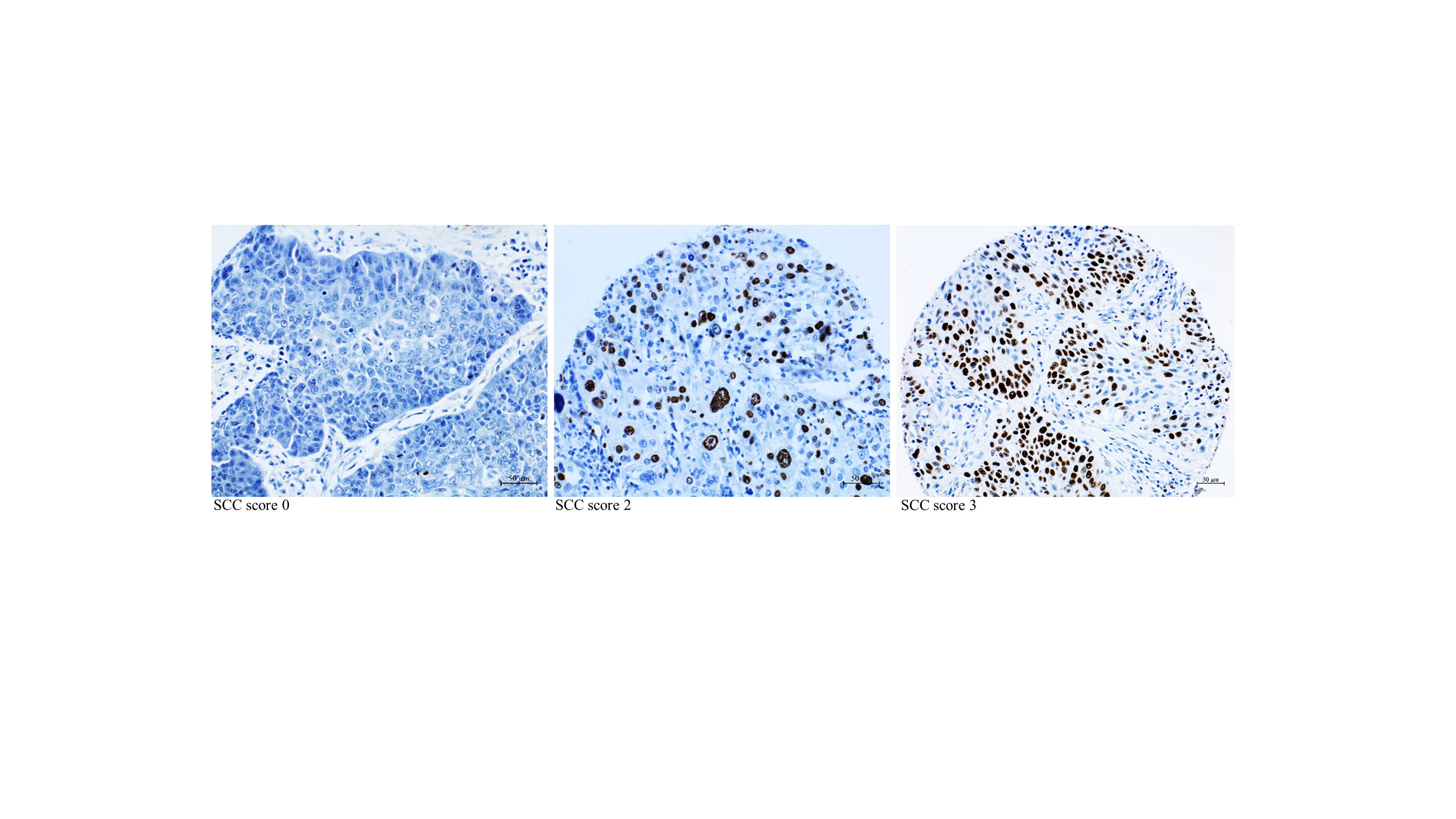

Supplement: Supplementary file 1 [file cancers-16-01170-s001.zip › Figure S4.tif]

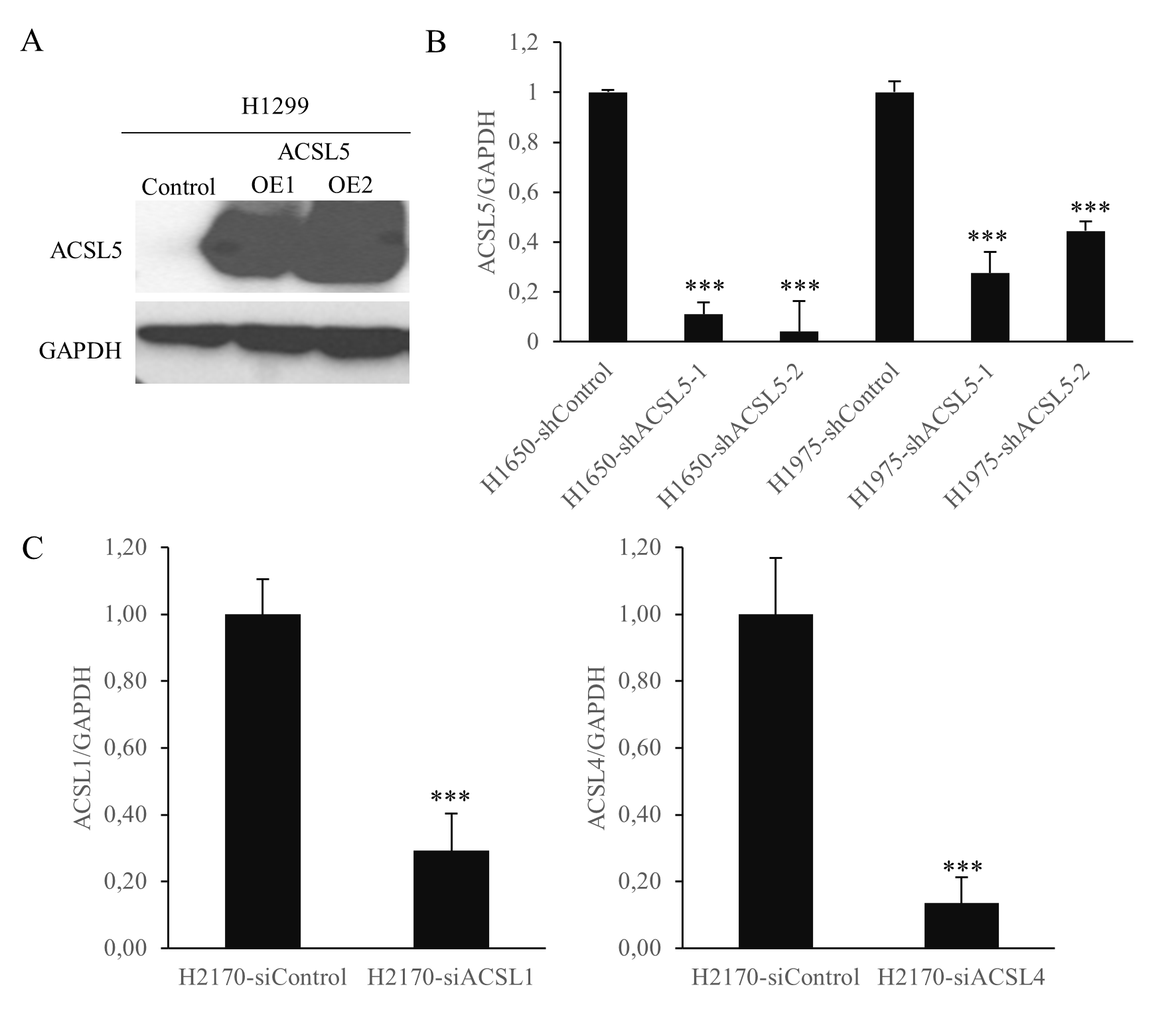

Supplement: Supplementary file 1 [file cancers-16-01170-s001.zip › Figure S5.tif]

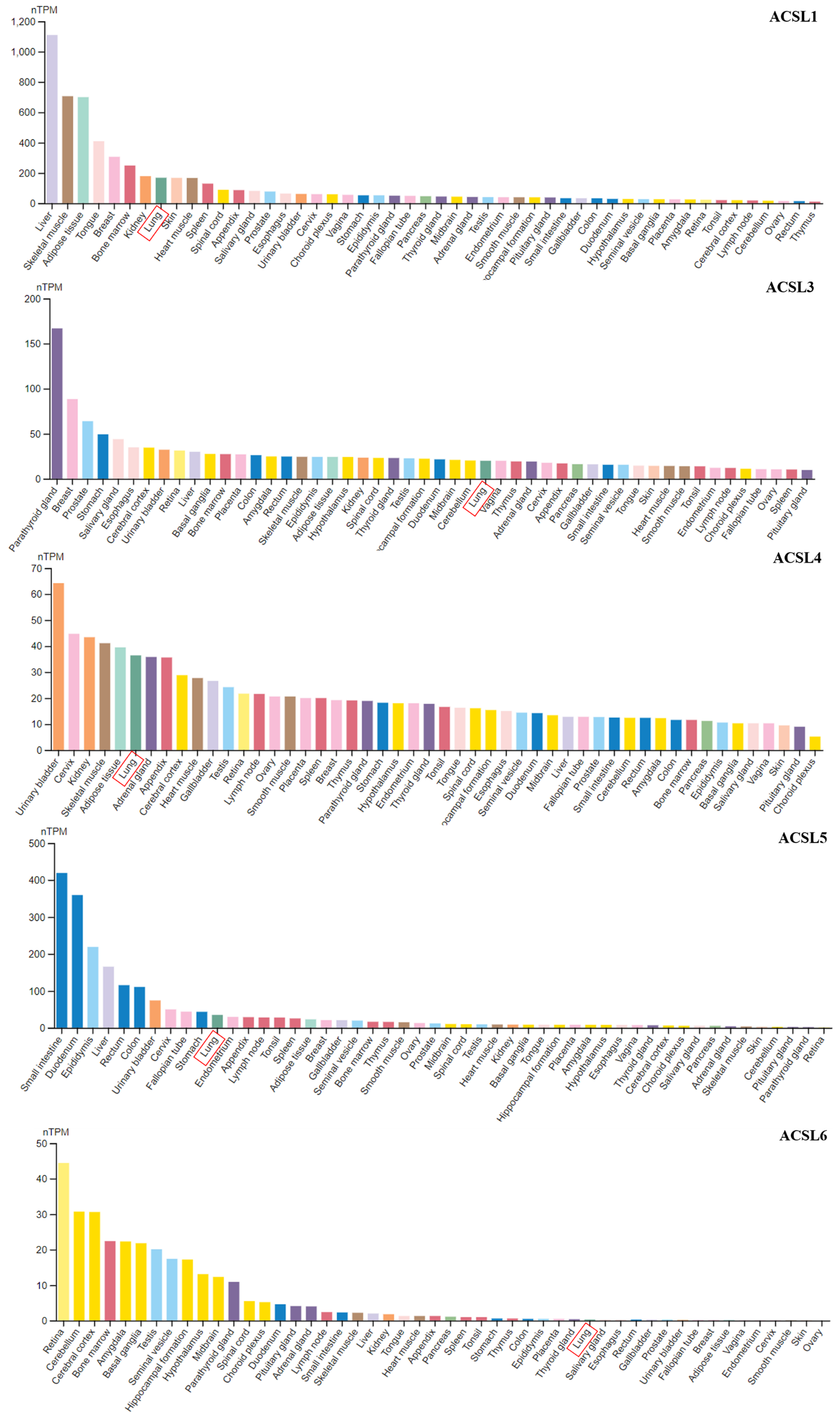

Supplement: Supplementary file 1 [file cancers-16-01170-s001.zip › Figure S6.TIF]

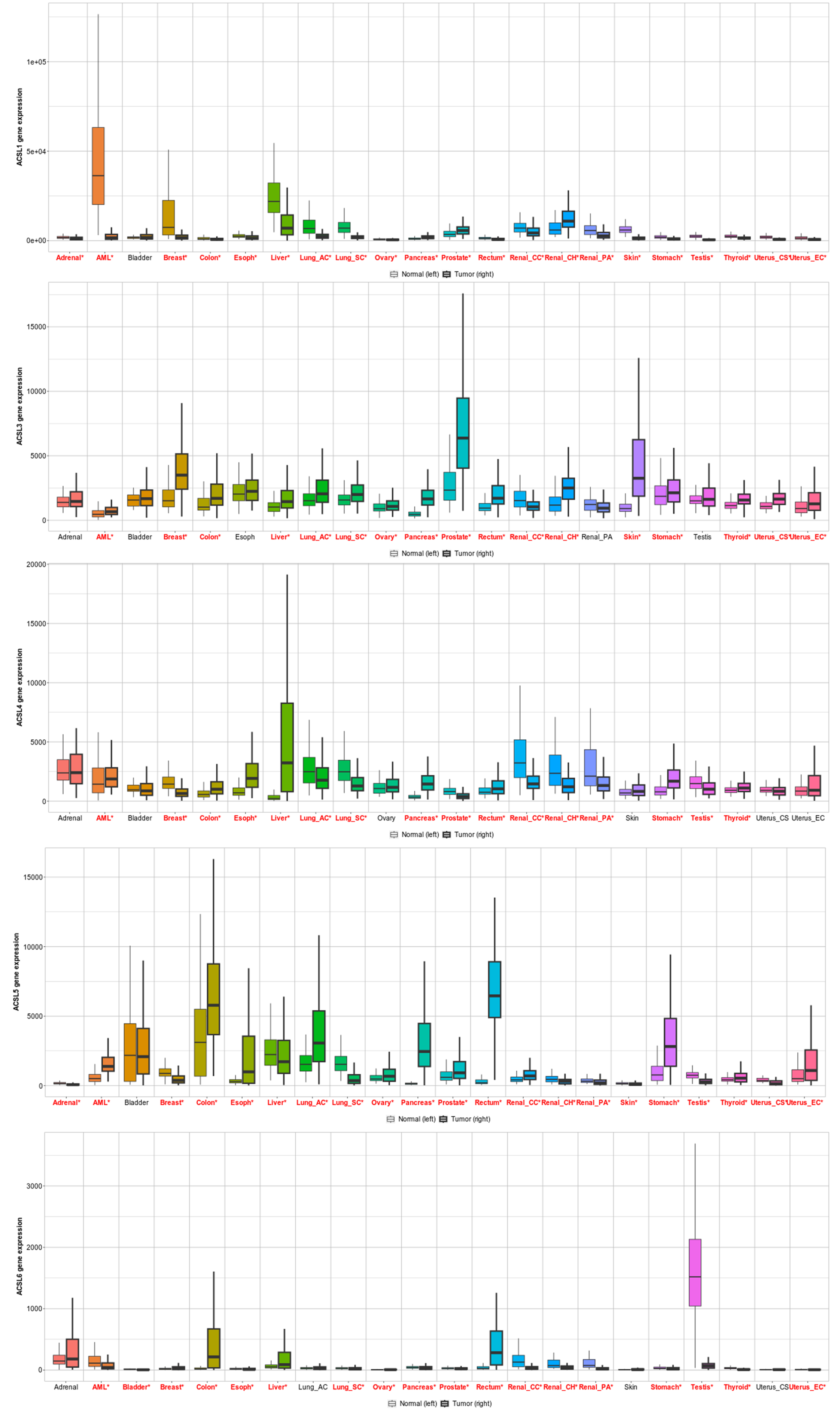

Supplement: Supplementary file 1 [file cancers-16-01170-s001.zip › Figure S7.TIF]

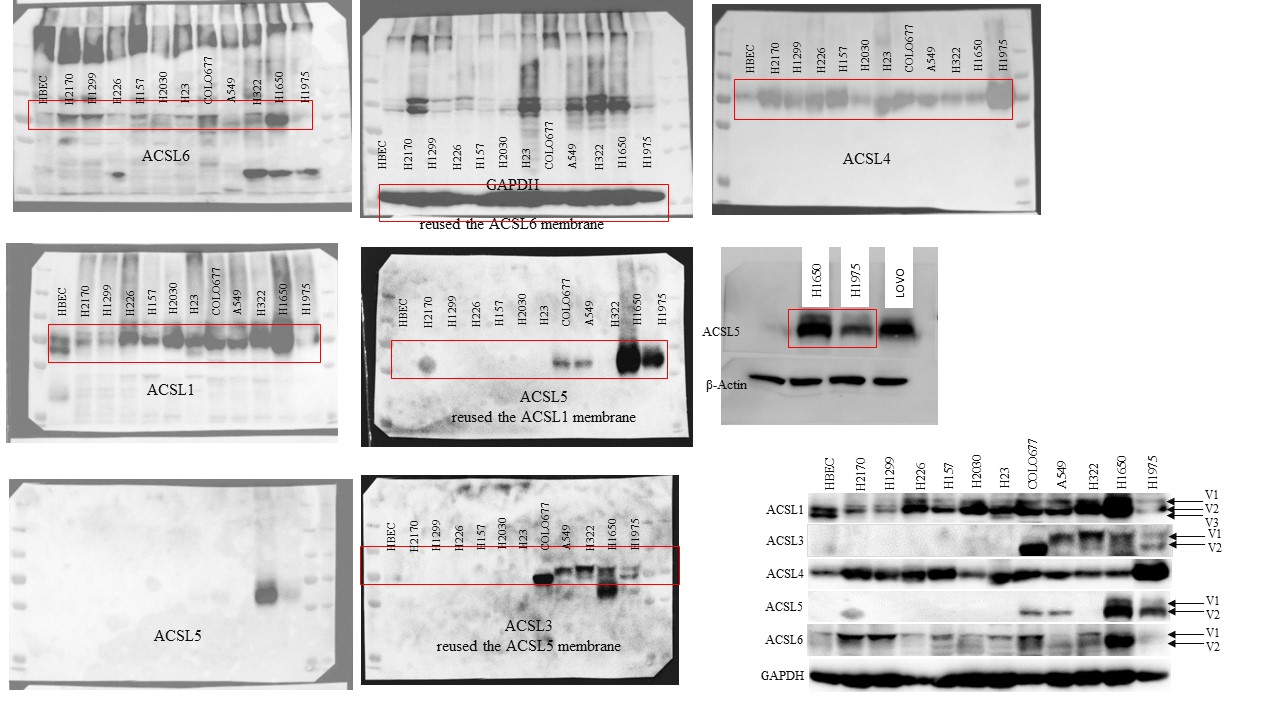

Supplement: Supplementary file 1 [file cancers-16-01170-s001.zip › Figure S8.jpg]

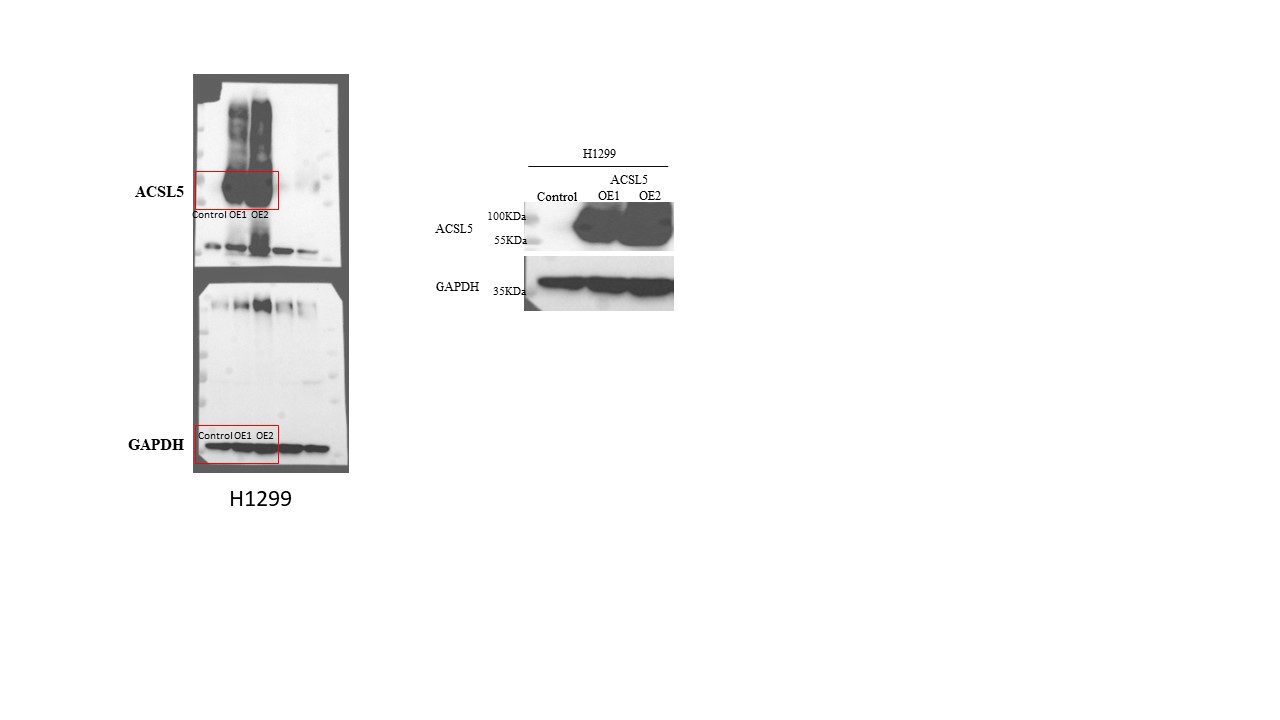

Supplement: Supplementary file 1 [file cancers-16-01170-s001.zip › Figure S9.jpg]
